# Enhancing Expert Panel Discussions in Pediatric Palliative Care: Innovative Scenario Development and Summarization With ChatGPT-4

**DOI:** 10.7759/cureus.38249

**Published:** 2023-04-28

**Authors:** Mohammed Almazyad, Fahad Aljofan, Noura A Abouammoh, Ruaim Muaygil, Khalid H Malki, Fadi Aljamaan, Abdullah Alturki, Tareq Alayed, Saleh S Alshehri, Abdullah Alrbiaan, Mohammed Alsatrawi, Hazar A Temsah, Fahad Alsohime, Ali A Alhaboob, Majed Alabdulhafid, Amr Jamal, Khalid Alhasan, Ayman Al-Eyadhy, Mohamad-Hani Temsah

**Affiliations:** 1 Pediatric Intensive Care Unit, Pediatric Department, College of Medicine, King Saud University, Riyadh, SAU; 2 Pediatric Intensive Care Unit, Pediatric Department, King Faisal Specialist Hospital & Research Centre, Riyadh, SAU; 3 Department of Family and Community Medicine, College of Medicine, King Saud University, Riyadh, SAU; 4 Medical Education Department, College of Medicine, King Saud University, Riyadh, SAU; 5 Department of Otolaryngology, College of Medicine, King Saud University, Riyadh, SAU; 6 Critical Care Department, College of Medicine, King Saud University, Riyadh, SAU; 7 Pediatric Critical Care Medicine, King Faisal Specialist Hospital & Research Centre, Riyadh, SAU; 8 Pediatric Intensive Care Unit, King Saud Medical City, Riyadh, SAU; 9 Critical Care Department, King Faisal Specialist Hospital & Research Centre, Riyadh, SAU; 10 Pediatric Critical Care Medicine, King Saud University Medical City, Riyadh, SAU; 11 Biomedical Engineering Department, Faculty of Electrical and Computer Engineering, Beirut Arab University, Beirut, LBN; 12 Pediatric Critical Care Department, King Saud University, Riyadh, SAU; 13 Department of Pediatrics, King Saud University, Riyadh, SAU; 14 Department of Family and Community Medicine, King Saud University, Riyadh, SAU; 15 Department of Pediatric Nephrology, King Saud University, Riyadh, SAU; 16 Pediatric Intensive Care Unit, King Saud University Medical City, Riyadh, SAU; 17 Pediatric Intensive Care Unit, Pediatric Department, King Saud University Medical City, Riyadh, SAU

**Keywords:** chat generative pre-trained transformer, picu multidisciplinary approach, pediatric palliative critical care, patient and family-centered care, human-ai scenario development, ethical considerations, do-not-resuscitate (dnr) conflicts discussion, medical conferences, expert panel discussions, artificial intelligence chatgpt-4

## Abstract

This study presents a novel approach to enhance expert panel discussions in a medical conference through the use of ChatGPT-4 (Generative Pre-trained Transformer version 4), a recently launched powerful artificial intelligence (AI) language model. We report on ChatGPT-4's ability to optimize and summarize the medical conference panel recommendations of the first Pan-Arab Pediatric Palliative Critical Care Hybrid Conference, held in Riyadh, Saudi Arabia. ChatGPT-4 was incorporated into the discussions in two sequential phases: first, scenarios were optimized by the AI model to stimulate in-depth conversations; second, the model identified, summarized, and contrasted key themes from the panel and audience discussions. The results suggest that ChatGPT-4 effectively facilitated complex do-not-resuscitate (DNR) conflict resolution by summarizing key themes such as effective communication, collaboration, patient and family-centered care, trust, and ethical considerations. The inclusion of ChatGPT-4 in pediatric palliative care panel discussions demonstrated potential benefits for enhancing critical thinking among medical professionals. Further research is warranted to validate and broaden these insights across various settings and cultures.

## Introduction

Pediatric palliative care is a complex and challenging field that involves ethical considerations, decision-making surrounding goals of care and code status, and the impact on families. In the pediatric critical care setting, determining who should decide end-of-life decisions - patients, parents, or healthcare providers - is a particularly challenging question in clinical practice. Multidisciplinary approaches involving experts in the field and community members in panel discussions or focus groups can enhance critical thinking among medical professionals using real or hypothetical cases [1].

Pediatric palliative care presents numerous challenges, including ethical considerations and the significant impact on families, necessitating effective communication and healthcare workers' (HCWs) decision-making processes to navigate these complexities [2]. With the rapidly evolving medical literature on artificial intelligence (AI) language models, many studies suggest various potential applications for the recently announced ChatGPT-4, the OpenAI’s (San Francisco, CA) most advanced chatbot that was developed to produce safer and more useful responses [3,4]. Launched on March 14, 2023, ChatGPT-4 is expected to perform at a level equivalent to that of humans on several professional and academic benchmarks [5]. In the context of pediatric palliative care, AI chatbots like ChatGPT-4 may offer unique benefits, such as facilitating complex medical discussions, promoting collaboration among healthcare professionals, and fostering patient and family-centered care. By addressing these challenges and integrating AI chatbots into the decision-making process, we hope to enhance the overall quality of care in this sensitive field.

Given the potential of AI technology to enhance panel discussions and expert knowledge sharing, this study seeks to explore the integration of ChatGPT-4 in such settings [6]. This study aims to investigate the efficacy of using ChatGPT-4 in panel discussions as an AI chatbot, to facilitate knowledge sharing and improve decision-making. The research seeks to describe the methodology used to integrate ChatGPT-4 into panel discussions and examine the benefits and limitations of using AI chatbots in such settings. Additionally, the study aims to explore the panel’s feedback on the accuracy and usefulness of ChatGPT-4's output and assess its impact on the panel’s final recommendations. Overall, this research aims to provide insights into the potential of AI chatbots in enhancing panel discussions and expert knowledge sharing. The contributions of this paper are twofold: first, it demonstrates the potential of AI technology in enhancing expert panel discussions and knowledge sharing; and second, it presents an innovative approach to optimizing and summarizing the outcomes of such discussions.

## Materials and methods

Study design

An integrated ChatGPT-4 qualitative research design was employed to explore the complexities of do-not-resuscitate (DNR) decisions in the pediatric intensive care unit (PICU) and palliative care context, including conflict resolution and decision-making authority, through focused group discussions with the PICU panel and attendees of a conference.

Setting

The inaugural Pan-Arab Pediatric Palliative Critical Care Hybrid Conference [7], which took place in Riyadh, Saudi Arabia, on the 16th of March 2023, was a groundbreaking event. Organized by the Saudi Critical Care Society Pediatric Chapter, this conference brought together medical professionals from across the world. Participants engaged in expert panel discussions that emphasized a thought-provoking central theme: "Who should be entrusted with making end-of-life decisions - patients, parents, or healthcare providers?" These critical conversations sought to explore the complex ethical dilemmas surrounding such decisions in pediatric palliative care.

Sample and recruitment

Four participants, known as "expert panelists," were purposefully selected from the attendees of the first Pan-Arab Pediatric Palliative Critical Care Hybrid Conference. These experts were chosen based on their extensive experience in pediatric palliative care, representing a variety of healthcare professions such as physicians, nurses, social workers, and other healthcare providers working in pediatric palliative care [7]. Invitations were sent via email to potential participants, and those who agreed to participate were included in the panel discussion groups. The panel was actively engaging in a one-hour focused discussion with the conference attendees.

The conversation addressed the multifaceted complexities surrounding DNR decisions, including topics such as emerging conflicts and their resolution, strategies for addressing the challenge of identifying the appropriate stakeholders and authorized decision-makers, and whether the primary team, PICU staff, the child, or the parents should be responsible for making these critical decisions. The panel focus group discussion was skillfully facilitated by FAJ, a pediatric intensivist, and the panelists (SA, AAR, and MHT), who are pediatric or adult intensivists, with contributions from content experts during the live discussions (MA, AAT, and TAA). Additionally, the panel encouraged frequent interactions with the attending audience, which consisted of approximately 70 participants, fostering an open and inclusive dialogue.

Intervention

**Figure 1:**
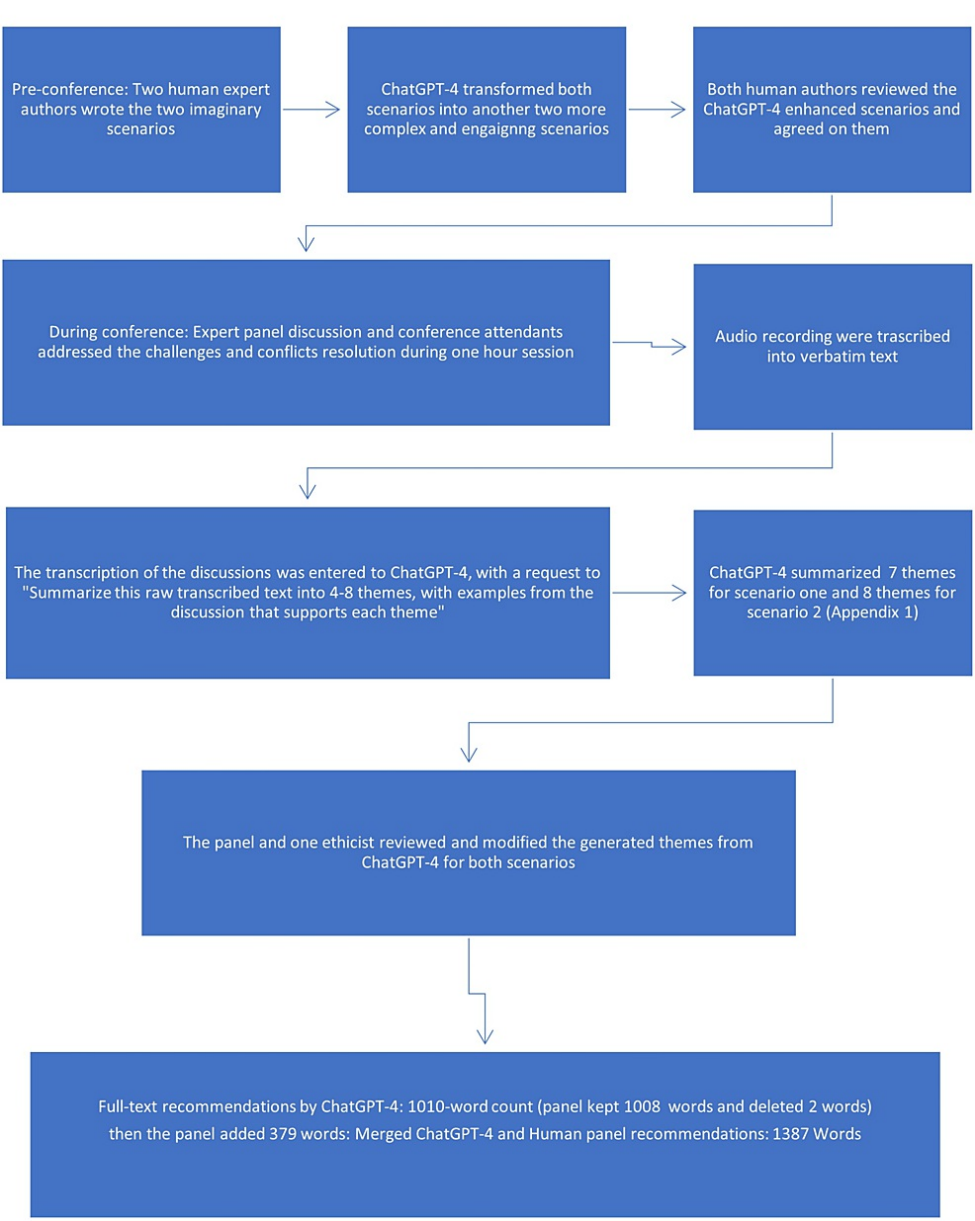
Flowchart describing the integration of ChatGPT-4 into complex expert panel discussions and generation of consensus medical conference recommendations.

To incorporate ChatGPT-4 into the panel discussions, the following steps were undertaken (Figure 1).

Scenario Development

The research team provided ChatGPT-4 with two human-written imaginary scenarios narrated about cystic fibrosis and severe meningococcal encephalitis (drafted by FAJ and MHT) related to DNR conflict resolution in pediatric critical and palliative care (Figure 1). ChatGPT-4 then transformed these human-written scenarios into more interactive and challenging case studies for the panelists to discuss.

Scenarios Panel Discussions

The expert panel discussed the scenario generated by ChatGPT-4 with an emphasis on conflict resolution and the patient’s and family’s wish for the perceived best delivery of care. Participants, both the expert panel and the conference attendees, were encouraged to engage in a dialogue about the cases of the scenarios, sharing their insights and experiences with one another. The discussions were audio recorded with permission from the team.

Scenario Presentation

The verbatim transcript from the panel discussion was transcribed and entered into ChatGPT-4. The scenarios' discussion outlines were revised based on the summary created with ChatGPT-4 and presented back to the expert panel on the day after the conference.

Data collection

The focused group discussions were audio-recorded and transcribed verbatim to ensure the accurate capture of participants’ contributions. During the discussions, a facilitator (FAJ) guided the conversation and encouraged the exchange of ideas and experiences. The discussions were conducted physically and lasted for 56 minutes.

Data Analysis and Summarization

Following the focused group discussions, we utilized ChatGPT-4 to analyze the transcribed data, being the first study to use ChatGPT-4 for analyzing qualitative data. This approach has been proven effective in the literature, as focus group discussions have been employed to evaluate theoretical scenarios [1]. The AI model identified key themes, patterns, and insights from the participants' conversations. ChatGPT-4 then generated a summarized version of the discussions, highlighting the main points and conclusions reached by the participants.

To ensure the validity and reliability of the findings, two members of the research team (FA and AJ) independently reviewed the summaries produced by ChatGPT-4. Discrepancies were resolved through discussion and consensus, ensuring that the final summary accurately reflected the participants' views and insights. Respondent validation was attained by introducing the recommendations produced from the analysis to four of the panel experts.

Ethical considerations

Prior to the panel discussions, informed consent was verbally obtained from all participants, and no incentives were offered to participants. They were informed of the purpose of the study, their right to withdraw at any time, and the use of ChatGPT-4 in data analysis and summarization. No identifiable information about the participants was shared with ChatGPT-4 at all. All data were anonymized to protect participants' privacy and maintain confidentiality. The IRB approval was obtained from King Saud University, Riyadh, Saudi Arabia (# 23/0203/IRB).

## Results

Case 1

The first case discussed an imaginary scenario that was drafted with ChatGPT-4 about potential conflict between the primary physician and other medical services in regard to DNR status for a complex case.

Pre-conference, the human author requested from ChatGPT-4: “Write a five-sentence scenario of eight-year-old cystic fibrosis, 10 kg, progressive especially last few months with several PICU admissions within four months. The multidisciplinary team denied him transplant due to poor weight, or ECMO, family is accepting the medical decision, but the primary physician insisted on full code, hoping to maximize his weight by TPN.”

ChatGPT-4 formulated the following scenario: “An eight-year-old child with cystic fibrosis, weighing only 10 kg, has been experiencing a rapid decline in health, particularly in the last few months, with multiple PICU admissions within a four-month period. The multidisciplinary team, after thorough evaluation, has denied the child a transplant due to their poor weight and deemed them not a suitable candidate for extracorporeal membrane oxygenation (ECMO). The family understands and accepts the medical team's decision, acknowledging the limitations of treatment options for their child. However, the primary physician remains hopeful and insists on a full code status, believing that initiating total parenteral nutrition (TPN) might help the child gain enough weight to qualify for further treatment options. Despite the differing opinions, the medical team and family continue to work together to determine the best course of action for the child's care.”

During the conference, the expert panel discussed the ChatGPT-4-generated scenario with emphasis on conflict resolution and the patient’s and family’s best possible care. The discussions were audio recorded with permission from the team.

Then, the verbatim transcript from the panel discussion was transcribed and entered into ChatGPT-4, with the following request: “The following, between quotes, are the raw transcripts of the panel discussions among PICU and adult ICU experts. Make four to eight themes, with examples from the discussion that supports each theme.”

In response, ChatGPT-4 generated the following output, which was examined and modified by the panelists and authors (Appendix) and is summarized in Table 1.

**Table 1:** Comparison of key themes in Scenario 1 and Scenario 2. HCWs: healthcare workers; TPN: total parenteral nutrition.

Case 1: Conflict between the primary team and other HCWs in end-of-life decision	Case 2: Conflict between HCWs and family for end-of-life decision
1. Consensus and collaboration: Medical team works together, avoiding conflicting messages, and addressing the primary physician's emotions	1. Patient prognosis and care consensus: Medical team reaches agreement on irreversible brain damage and ineffectiveness of aggressive treatment
2. Patient and family-centered care: Considering the child's perspective and family's understanding, providing consistent information	2. Family's emotional state and denial: Recognizing grief, approaching the situation with empathy and understanding
3. Balancing treatment options and end-of-life care: Debating the viability of TPN and full support, presenting the decision as a therapeutic recommendation	3. Communication with the family: Avoiding certain terminology, addressing unrealistic expectations, empathy, and trust-building techniques
4. Trust and transparency: Open communication, addressing concerns, providing accurate information to family and primary physician	4. Maintaining trust and alignment among medical professionals: Pre-counseling, ongoing discussions, and consistent information delivery
5. Data-driven decision-making: Using data to support decisions, informing discussions with an evidence-based approach	5. Cultural considerations: Sensitivity to family's cultural/religious beliefs, avoiding generalization, and personalized patient care
6. Ethical considerations: Informed consent, respecting professional expertise, advocating for personal perspectives	6. Miracle’s perceptions and ethical considerations: Acknowledging religious miracles, assessing applicability based on patient's condition, respecting religious beliefs
7. Conflict resolution: Addressing disagreements, soliciting opinions from silent parties, creating a collaborative environment	7. Resource allocation and care cost: Balancing patient needs with healthcare system realities, addressing financial burden
8. Assessing other HCWs' readiness for decision-making: Observing verbal and nonverbal cues, addressing concerns, and adjusting communication strategies based on emotional state and acceptance level	8. Assessing the family's readiness for decision-making: Observing verbal and nonverbal cues, adjusting communication strategies based on emotional state and acceptance level

“After analyzing the raw transcript, the following themes and examples can be identified:

1. Consensus and collaboration: It is crucial for the medical team to achieve a consensus among themselves, including primary physicians, intensivists, nurses, and the rest of the close healthcare providers, to avoid sending conflicting messages to the family. For example, one panelist emphasized the importance of dealing with the primary physician's emotions, as they may be emotionally attached to the child, and their opinion, influenced by this emotional bonding, can impact the family's decision-making.

The primary physician in some cultures may behave paternalistically to the extent of feeling sad and depressed near end-of-life decisions. This emotional state of the primary physician was highlighted during the discussion, and it underscores the need to approach them with care, similar to how the family is approached. This unique description of the primary physician's role is an interesting phenomenon in some medical communities, and acknowledging this complexity does not imply an obvious "right" choice, but rather emphasizes the importance of understanding and navigating the conflict.

2. Patient and family-centered care: The panelists discussed the need to consider the child's perspective and the family's understanding of the situation. They mentioned the importance of avoiding confusion for the family by ensuring that they receive consistent information from the medical team.

3. Balancing treatment options and end-of-life care: The panelists debated whether TPN and full support are viable options for the child, considering the child's weight, the potential for improvement, and potential risks and complications. One panelist suggested that TPN could be tried, but the discussion about DNR should not be delayed. If the medical opinion strongly supports a DNR order, then this must be presented as an explicit recommendation to the parents, rather than as one of several equally viable management options, to alleviate the burden of decision-making and to ensure an informed decision.

4. Trust and transparency: Maintaining trust within the healthcare system and between the medical team and the family is vital. The panelists highlighted the importance of open communication, addressing concerns, and providing accurate information to both the family and the primary physician.

5. Data-driven decision-making: The panelists suggested using data to support their decisions, such as asking the primary physician about their experience with similar cases and the likelihood of achieving the targeted weight gain for the child. This can help to inform the discussion and facilitate a more evidence-based approach.

6. Ethical considerations: The panelists emphasized the importance of informed consent, ensuring that the family fully understands the potential risks and benefits of any proposed interventions. They also discussed the need to respect professional expertise and opinions while still advocating for their own perspectives.

7. Conflict resolution: The panelists emphasized the importance of addressing disagreements and conflicts within the medical team and highlighted the value of involving additional expertise from the same specialty (e.g., pulmonologists) to assess the impact of specific treatments, such as TPN, which in the case scenario, was the primary reason the primary MD was not accepting a DNR order. Moreover, it is crucial to engage other healthcare providers or family members who may have been "silent" during discussions, as they can play a significant role in influencing parental decisions. Fostering a more collaborative environment can facilitate better decision-making for the child's care. Involving a second opinion from the same specialty can provide a broader understanding of complex cases and offer valuable insights to both the treating team and the family.

8. Assessing other healthcare providers' readiness for end-of-life decision-making discussions: The panelists acknowledged the importance of considering the emotional and psychological readiness of other healthcare providers involved in the child's care when engaging in end-of-life decision-making discussions. Recognizing and addressing any hesitations, concerns, or attachments that may impact their decision-making process can help ensure that these providers contribute effectively to the conversation and maintain a consistent message for the family. This may include offering support, providing additional information, or facilitating discussions among the healthcare team to address any discrepancies and establish a unified approach.”

Case 2

The second case discussed an imaginary scenario that was drafted with ChatGPT-4 about potential conflict between the PICU team and a patient’s family regarding DNR status.

Pre-conference, the human author requested from ChatGPT-4: “Write a five-sentence scenario of a previously healthy nine-year-old child with severe meningoencephalitis that progressed within 12 hours of PICU admission to severe brain edema despite neuroprotective measures, and progressed to herniation. On PICU day six, the PICU, neurology, and neurosurgery, all agreed about the irreversible severe brain insult, but the family was in denial and refusing DNR.”

ChatGPT-4 formulated the following scenario: “A previously healthy nine-year-old child was admitted to the pediatric intensive care unit (PICU) with severe meningoencephalitis. Despite the best efforts of the medical team, including neuroprotective measures, the child's condition rapidly deteriorated within 12 hours of admission, resulting in severe brain edema and subsequent brain herniation. By PICU day six, the PICU team, neurologists, and neurosurgeons all agreed that the child had suffered irreversible severe brain damage. However, the family, struggling to come to terms with the situation, remained in denial about the severity of their child's condition and refused to consider a do-not-resuscitate (DNR) order, insisting on continued aggressive treatment.”

After the expert panel discussed this second scenario during the conference, we uploaded the transcribed discussion to ChatGPT-4 with the following request: “For the imaginary scenario of a previously healthy nine-year-old child who was admitted to the pediatric intensive care unit (PICU) with severe meningoencephalitis, the panel of intensivists had the following panel discussions, between quotes. Summarize this raw transcribed text into four to eight themes, with examples from the discussion that supports each theme.”

In response, ChatGPT-4 generated the following output, which was also reviewed and finalized by the panelists (Table 1).

“After reviewing the raw transcribed text, here are the four to eight themes with examples from the discussion that support each theme:

1. Patient prognosis and care consensus: The medical team, including neurologists and neurosurgeons, reached a consensus that the patient had irreversible brain damage due to severe meningoencephalitis. They also discussed the ineffectiveness of aggressive treatment that might include performing CPR or intubation and the potential consequences of continuing such treatment.

2. Family's emotional state and denial: The family struggled to accept the severity of their child's condition and remained in denial, refusing to consider a DNR and insisting on continuing aggressive treatment. The panel recognized the family's grief and the need to approach the situation with empathy and understanding.

3. Communication with the family: The panel discussed various strategies for communicating with the family, including avoiding certain terminology (e.g., CPR), not putting the family in a position to take responsibility for the decision, addressing unrealistic expectations, and not giving false hope, and using empathy and trust-building techniques. They emphasized the importance of multiple meetings to gradually help the family understand and accept the situation. It was stressed to value the family’s religious background and the need for a religious mediator to support the family and explore applicable religious beliefs and principles to support the decision of DNR.

4. Maintaining trust and alignment among medical professionals: The panel highlighted the need to maintain trust among the medical team and ensure that all team members were on the same page. This included pre-counseling and ongoing discussions with the family to make sure they received consistent information from the team.

5. Cultural considerations: The panel mentioned the importance of understanding cultural context when discussing the patient's prognosis, such as not verbalizing “no hope” or “futile intervention” directly and being sensitive to the family's cultural/religious beliefs and values. The panel emphasized the need for healthcare providers to avoid generalizing or stereotyping based on cultural context and to provide personalized patient care, as not all patients or families share the same cultural beliefs and expectations. Therefore, it is important to recognize that some may want to know specific information about their prognosis.

In addition, the expert panelists highlighted the potential conflict with the Islamic cultural view, where the terms “no hope” and “futility” may be interpreted as a lack of faith in Allah's capacity and capabilities. They suggested avoiding these terms and being mindful of how they may impact the family's trust in the healthcare provider. The PICU consultant noted that expressions of hope and faith in God are often shared by the family and healthcare provider, but it is important to avoid referring to a specific pathological condition that may not be in line with the reality of the situation. By recognizing the cultural and religious beliefs and expectations of the family, healthcare providers can engage in open and honest communication that is sensitive to the family's values and beliefs while providing personalized patient care.

6. Miracle perceptions and ethical considerations: The panel also deliberated on the ethical considerations surrounding families' beliefs in the potential for miraculous recoveries. They discussed instances where families referred to cases they had heard about, wherein patients experienced unexpected full recoveries from prolonged comatose illnesses. The panel suggested that acknowledging the concept of religious miracles could be a reasonable approach to gaining the trust of families while also ensuring the applicability of such miracle events is carefully assessed based on the current patient's condition. This approach demonstrates respect for the families' religious beliefs and fosters a supportive environment for open communication.

7. Resource allocation and care cost: The panel discussed the implications of scarce resource allocation and the associated costs of prolonged PICU stays. They acknowledged that addressing the financial burden of extended hospitalization and managing limited resources are essential aspects of pediatric palliative care decision-making. Balancing the needs of the patients with the realities of healthcare systems is a challenging task that requires careful consideration.

8. Assessing the family's readiness for decision-making: The panel discussed gauging the family's readiness for decision-making, such as by observing their verbal and nonverbal cues, asking about their expectations, and adjusting communication strategies based on their emotional state and acceptance level. For example, if a family appears emotionally distressed during the conversation, slow down and give them more time to grieve.”

Table 1 compares key themes from expert panel discussions in Scenarios 1 and 2. Scenario 1 addresses conflicts among HCWs in end-of-life decision-making, while Scenario 2 focuses on conflicts between HCWs and the patient's family. The table reveals similarities and differences in themes, emphasizing unique challenges and considerations in each scenario, including consensus, communication, ethics, and assessing readiness for decision-making among HCWs and family members.

## Discussion

The panel discussions offered important insights into the challenges of DNR decisions in the PICU setting, such as conflict resolution and complex pediatric palliative care. Key themes emerged, meriting further investigation. Using the advanced ChatGPT-4 AI chatbot streamlined expert panel discussions' preparation and facilitated efficient summarization of opinions into well-defined themes.

Firstly, the importance of consensus and collaboration among healthcare professionals was emphasized, as it is crucial to present a unified message to the family to avoid confusion, miscommunication, and eventually distrust [8,9]. Open dialogue and understanding within the medical team, including addressing disagreements and emotions, are essential to ensure the best patient care [10].

Secondly, the discussions highlighted the necessity of patient and family-centered care, emphasizing the need for empathy, understanding, and effective communication with the family [11]. This includes addressing the family's emotional state, like denial, as well as respecting their cultural beliefs and preferences. For example, the integration of palliative care into the routine care of children and adolescents with cancer has resulted in improved outcomes for patients and their families [12]. Gradual and ongoing communication with the family can help them better understand the medical situation and make coherent informed decisions [13].

Balancing treatment options and end-of-life care emerged as another important theme, with panelists debating the viability and ethical implications of various interventions [14-16], such as TPN in the case of the cystic fibrosis patient. This highlights the significance of a comprehensive decision-making process that incorporates medical evidence and professional expertise, as well as the patient's and family's goals and preferences. By integrating these elements, healthcare providers can facilitate more effective discussions and ultimately determine the most appropriate course of action for the patient. Providing efficient management of symptoms during end-of-life care can enable patients to go through the process of dying with safety, dignity, and comfort [17].

Considering the patient's known wishes, when available, is crucial in respecting their autonomy and right to self-determination, even though this may not always be feasible in a pediatric context. Equally important in ethical decision-making is supporting the family as they navigate decision-making processes and cope with their grief. Furthermore, collaborating with the patient's primary physician demonstrates professional respect and fosters partnership among colleagues while allowing the provider to address their own professional grief in the face of losing a patient. The family-centered multidisciplinary team approach helps in navigating this challenging period while observing the ethical principles, such as respect for people's rights and dignity and the principle of non-maleficence [18].

Following the panel and conference attendees’ discussions, the use of ChatGPT-4 helped to summarize the extensive human-human discussions, rapidly and reliably, with the AI generating a structured summarization in these complex medical situations that contribute to prolonged hospitalization and resource utilization [19-21]. We hereby demonstrated for the first time in the medical literature integrating ChatGPT-4 in re-generating more interactive and challenging scenarios [22-24], then using the same AI chatbot to analyze and summarize the panel discussion transcripts to provide a meaningful summary of key themes, insights, and expert consensus recommendations, to be shared with the medical literature. This highlights the potential for AI to play an increasingly significant role in healthcare, augmenting the expertise of healthcare professionals and improving patient care outcomes [25-27].

Limitations

Although the panel discussions and ChatGPT-4 usage provided valuable insights and an engaging platform, several limitations must be acknowledged. The panel discussions took place at a specific conference with a select group of regional experts, potentially limiting the generalizability of findings to other contexts or healthcare settings with different cultural, social, and healthcare contexts. Expert panelists may have been influenced by personal experiences, beliefs, and professional backgrounds, introducing potential biases. Qualitative research design limitations include subjectivity, as different researchers might identify alternative themes or place varying emphasis on certain aspects. Moreover, the focused group discussions and qualitative analysis do not provide quantitative data to support or measure the effectiveness of identified themes in practice. Future research, including quantitative and mixed-methods studies, could help validate and expand upon these panel discussions' findings.

Limitations related to ChatGPT-4 should be considered. Although ChatGPT-4 was successful in generating scenarios and summarizing panel discussions, it is important to note that AI-generated content can sometimes be biased, inaccurate, or lack context [4,6,28]. The AI model's output should be carefully reviewed and validated by experts before drawing conclusions or making decisions based on its analysis, and more research is warranted [29]. Although the methodology employed to analyze the transcripts generated from the focus group discussion was not explicitly detailed, it successfully yielded logical, topic-related themes.

In addition to the limitations mentioned, we also highlight the following points.

Limited Training Data

ChatGPT-4's training data may not have included a comprehensive representation of the pediatric palliative care literature or perspectives from different cultural backgrounds. This limitation could impact the AI model's ability to generate relevant and contextually appropriate content, potentially introducing biases or inaccuracies in the generated scenarios and summaries.

AI Model Adaptation

The dynamic nature of medical knowledge and advancements in pediatric palliative care may not be immediately reflected in the AI model's understanding. Therefore, it is essential to continually update and refine the AI model to ensure that it remains relevant and accurate.

Ethical Considerations

The use of AI in sensitive fields like pediatric palliative care raises ethical concerns that must be observed, including patient privacy, informed consent, and potential misuse of AI-generated content. These concerns need to be thoroughly addressed and managed to ensure the responsible use of AI technology in healthcare settings [30].

Dependence on AI

Overreliance on AI-generated content could potentially lead future HCWs to reduced critical thinking and decision-making abilities, as they may become overly dependent on the technology. It is crucial to strike a balance between leveraging AI's benefits and maintaining human expertise in the decision-making process.

Despite these limitations, the panel discussions and the use of ChatGPT-4 have provided valuable insights into DNR conflict resolution and the potential role of AI in pediatric palliative care. In terms of future scope, further research could focus on using ChatGPT-4 in different healthcare settings and contexts to determine its effectiveness in enhancing expert panel discussions and knowledge sharing. The integration of quantitative and mixed-methods studies could also help to validate and expand upon the findings of this study. Finally, exploring the potential of AI in other areas of health care could lead to innovative solutions for improving patient care in challenging situations.

## Conclusions

In conclusion, our study highlights the potential of AI language chatbots, such as ChatGPT-4, to augment expert panel discussions and knowledge exchange in medical conferences. By utilizing AI-improved scenarios and summarization, we facilitated an interactive exploration of intricate medical discussions and conflict resolution and identified key themes focusing on communication, collaboration, patient and family-centered care, trust, and ethics. These findings hold substantial implications for clinical practice, as they emphasize the crucial role of effective communication, collaboration, and patient-centered care in pediatric palliative care decision-making. While small workshops employing a collaborative approach can aid in developing solutions by addressing participants' concerns and fostering a deeper understanding of complex topics, it is crucial to remain aware of potential biases or issues within AI-generated content. Meticulous validation of AI-generated outputs by human experts is necessary to ensure that decisions are based on accurate information. We suggest further research to validate and build upon these insights, with more exploration and research across diverse settings and cultures.

## Appendices

Original ChatGPT-4-generated output (regular font) and the panelists added content *(italic font*) summaries for case scenarios 1 and 2.

Case 1

“After analyzing the raw transcript, the following themes and examples can be identified:

1. Consensus and collaboration: It is crucial for the medical team to achieve a consensus among themselves, including primary physicians, intensivists, nurses, *and the rest of the close healthcare providers, *to avoid sending conflicting messages to the family. For example, one panelist emphasized the importance of dealing with the primary physician's emotions, as they may be emotionally attached to the child, and their opinion, influenced by this *emotional bonding*, can impact the family's decision-making.

The primary physician in some cultures may behave paternalistically to the extent of feeling sad and depressed near end-of-life decisions. This emotional state of the primary physician was highlighted during the discussion, and it underscores the need to approach them with care, similar to how the family is approached. This unique description of the primary physician's role is an interesting phenomenon in some medical communities, and acknowledging this complexity does not imply an obvious "right" choice, but rather emphasizes the importance of understanding and navigating the conflict.

2. Patient and family-centered care: The panelists discussed the need to consider the child's perspective and the family's understanding of the situation. They mentioned the importance of avoiding confusion for the family by ensuring that they receive consistent information from the medical team.

3. Balancing treatment options and end-of-life care: The panelists debated whether TPN and full support are viable options for the child, considering the child's weight, the potential for improvement, and potential risks and complications. One panelist suggested that TPN could be tried, but the discussion about DNR should not be delayed. *If the medical opinion strongly supports a DNR order, then this must be presented as an explicit recommendation to the parents, rather than as one of several equally viable management options, to alleviate the burden of decision-making and to ensure an informed decision.*

4. Trust and transparency: Maintaining trust within the healthcare system and between the medical team and the family is vital. The panelists highlighted the importance of open communication, addressing concerns, and providing accurate information to both the family and the primary physician.

5. Data-driven decision-making: The panelists suggested using data to support their decisions, such as asking the primary physician about their experience with similar cases and the likelihood of achieving *the targeted* weight gain for the child. This can help to inform the discussion and facilitate a more evidence-based approach.

6. Ethical considerations: The panelists emphasized the importance of informed consent, ensuring that the family fully understands the potential risks and benefits of any proposed interventions. They also discussed the need to respect professional expertise and opinions while still advocating for their own perspectives.

7. Conflict resolution: The panelists emphasized the importance of addressing disagreements and conflicts within the medical team and highlighted the value of involving additional expertise from the same specialty (e.g., pulmonologists) to assess the impact of specific treatments, such as TPN, which in the case scenario, was the primary reason the primary MD was not accepting a DNR order.* Moreover, it is crucial to engage other healthcare providers or family members who may have been "silent" during discussions, as they can play a significant role in influencing parental decisions. Fostering a more collaborative environment can facilitate better decision-making for the child's care. Involving a second opinion from the same specialty can provide a broader understanding of complex cases and offer valuable insights to both the treating team and the family.*

8. Assessing other healthcare providers' readiness for end-of-life decision-making discussions: The panelists acknowledged the importance of considering the emotional and psychological readiness of other healthcare providers involved in the child's care when engaging in end-of-life decision-making discussions. Recognizing and addressing any hesitations, concerns, or attachments that may impact their decision-making process can help ensure that these providers contribute effectively to the conversation and maintain a consistent message for the family. This may include offering support, providing additional information, or facilitating discussions among the healthcare team to address any discrepancies and establish a unified approach.”

Case 2

“After reviewing the raw transcribed text, here are the 4-7 themes with examples from the discussion that support each theme:

1. Patient prognosis and care consensus: The medical team, including neurologists and neurosurgeons, reached a consensus that the patient had irreversible brain damage due to severe meningoencephalitis. They also discussed the ineffectiveness of aggressive treatment *that might include performing CPR or intubation *and the potential consequences of continuing such treatment.

2. Family's emotional state and denial: The family struggled to accept the severity of their child's condition and remained in denial, refusing to consider a DNR and insisting on continuing aggressive treatment. The panel recognized the family's grief and the need to approach the situation with empathy and understanding.

3. Communication with the family: The panel discussed various strategies for communicating with the family, including avoiding certain terminology (e.g., CPR), not putting the family in a position to take responsibility for the decision, *addressing unrealistic expectations *not giving false hope, and using empathy and trust-building techniques. They emphasized the importance of multiple meetings to gradually help the family understand and accept the situation. *It was stressed to value the family’s religious background and the need for a religious mediator to support the family and explore applicable religious beliefs and principles to support the decision of DNR.*

4. Maintaining trust and alignment among medical professionals: The panel highlighted the need to maintain trust among the medical team and ensure that all team members were on the same page. This included pre-counseling and ongoing discussions with the family to make sure they received consistent information from the team.

5. Cultural considerations: The panel mentioned the importance of understanding cultural context when discussing the patient's prognosis, such as not verbalizing “no hope” or “futile intervention” directly and being sensitive to the family's cultural/religious beliefs and values. The panel emphasized the need for healthcare providers to avoid generalizing or stereotyping based on cultural context and to provide personalized patient care, as not all patients or families share the same cultural beliefs and expectations. Therefore, it is important to recognize that some may want to know specific information about their prognosis.

*In addition, the expert panelists highlighted the potential conflict with the Islamic cultural view, where the terms “no hope”*​​​​​​​* and “*​​​​​​​*futility”​​​​​​​ may be interpreted as a lack of faith in Allah's capacity and capabilities. They suggested avoiding these terms and being mindful of how they may impact the family's trust in the healthcare provider. The PICU consultant noted that expressions of hope and faith in God are often shared by the family and healthcare provider, but it is important to avoid referring to a specific pathological condition that may not be in line with the reality of the situation. By recognizing the cultural and religious beliefs and expectations of the family, healthcare providers can engage in open and honest communication that is sensitive to the family's values and beliefs while providing personalized patient care.*

6. Miracle perceptions and ethical considerations: The panel also deliberated on the ethical considerations surrounding families' beliefs in the potential for miraculous recoveries. They discussed instances where families referred to cases they had heard about, wherein patients experienced unexpected full recoveries from prolonged comatose illnesses. *The panel suggested that acknowledging the concept of religious miracles could be a reasonable approach to gain the trust of families while also ensuring the applicability of such miracle events is carefully assessed based on the current patient's condition. This approach demonstrates respect for the families' religious beliefs and fosters a supportive environment for open communication.*

7. Resource allocation and care cost: The panel discussed the implications of scarce resource allocation and the associated costs of prolonged PICU stays. They acknowledged that addressing the financial burden of extended hospitalization and managing limited resources are essential aspects of pediatric palliative care decision-making. Balancing the needs of the patients with the realities of healthcare systems is a challenging task that requires careful consideration.

8. Assessing the family's readiness for decision-making: The panel discussed gauging the family's readiness for decision-making, such as by observing their verbal and nonverbal cues, asking about their expectations, and adjusting communication strategies based on their emotional state and acceptance level. *For example, if a family appears emotionally distressed during the conversation, slow down and give them more time to grieve.”*
